# Latent profile analysis in frontotemporal lobar degeneration and related disorders: clinical presentation and SPECT functional correlates

**DOI:** 10.1186/1471-2377-7-9

**Published:** 2007-05-16

**Authors:** Barbara Borroni, Mario Grassi, Chiara Agosti, Barbara Paghera, Antonella Alberici, Monica Di Luca, Daniela Perani, Alessandro Padovani

**Affiliations:** 1 Center for Aging Brain and Dementia, Department of Neurology, University of Brescia, Brescia, Italy; 2 Department of Health Sciences, Section of Medical Statistics & Epidemiology, University of Pavia, Pavia, Italy; 3 Nuclear Medicine, Brescia Hospital, Brescia, Italy; 4 Centre of Excellence for Neurodegenerative Disorders and Department of Pharmacological Sciences, University of Milan, Milan, Italy; 5 Vita Salute San Raffaele University and IRCCS H San Raffaele, IBFN-CNR, Milan, Italy

## Abstract

**Background:**

Frontotemporal Lobar Degeneration (FTLD) thus recently renamed, refers to a spectrum of heterogeneous conditions. This same heterogeneity of presentation represents the major methodological limit for the correct evaluation of clinical designation and brain functional correlates. At present, no study has investigated clinical clusters due to specific cognitive and behavioural disturbances beyond current clinical criteria.

The aim of this study was to identify clinical FTLD presentation, based on cognitive and behavioural profile, and to define their SPECT functional correlations.

**Methods:**

Ninety-seven FTLD patients entered the study. A clinical evaluation and standardised assessment were preformed, as well as a brain SPECT perfusion imaging study. Latent Profile Analysis on clinical, neuropsychological, and behavioural data was performed. Voxel-basis analysis of SPECT data was computed.

**Results:**

Three specific clusters were identified and named "pseudomanic behaviour" (LC1), "cognitive" (LC2), and "pseudodepressed behaviour" (LC3) endophenotypes. These endophenotypes showed a comparable hypoperfusion in left temporal lobe, but a specific pattern involving: medial and orbitobasal frontal cortex in LC1, subcortical brain region in LC2, and right dorsolateral frontal cortex and insula in LC3.

**Conclusion:**

These findings provide evidence that specific functional-cluster symptom relationship can be delineated in FTLD patients by a standardised assessment. The understanding of the different functional correlates of clinical presentations will hopefully lead to the possibility of individuating diagnostic and treatment algorithms.

## Background

Frontotemporal Lobar Degeneration (FTLD) refers to a spectrum of heterogeneous conditions [1]. The disease designation and the disease concept were renamed in the past few years as different clinical criteria have been evolved over time. Until now, all these syndromes have been recognised to present with cognitive and language disturbances, personality changes and behavioural symptoms, including different clinical and anatomical conditions. Primary Progressive Aphasia (PPA), also called progressive nonfluent aphasia (PNFA), typically occurs with dominant frontotemporal atrophy [2,3]. These patients do not initially demonstrate behavioural abnormality. If the dominant temporal lobe is involved, FTLD presents with anomia, and the result is often impairment in language comprehension and loss of meaning, called Semantic Dementia (SD), which is often associated with behavioural abnormalities [1]. When behavioural abnormalities are the presenting feature, usually with nondominant temporal and frontal atrophy, the label "behavioural variant" (bvFTD) is used [4]. The recognition of a genetic and clinical overlap of FTLD with Corticobasal Degeneration (CBD) and Progressive Supranuclear Palsy (PSP) suggested the inclusion of these relatively rare conditions under the same nosographic label of FTLD [5,6].

Therefore, different presentations were separated into independent entities, creating a terminological proliferation and multiplying rare diseases. The classification of these diverse conditions remains controversial, some groups perceive FTLD as a unitary continuum whereas others describe more discrete syndromes [5,7].

Pathological fractionation did not lag behind, as histochemistry and molecular biology yielded new features [8]. Because of excessive deposits of hyperphosphorylated tau in affected neurons and glia, the underlying pathology of FTLD was regarded as a tauopathy. Paradoxically, the general label of tauopathy has been expanded to include cases in which tau was not found in the brain, and the vast majority of FTLD pathology is actually recognized by ubiquitin positive and tau negative inclusions.

FTLD conditions, however, have more in common than previously recognised, and the term Pick's Complex was chosen to signify their cohesion [5].

In fact, an integrative approach based on clinicopathological correlation suggests that not only the bvFTD, SD, and PNFA overlap clinically and pathologically, but the extrapyramidal component, i.e. CBD and PSP, should be considered part of an overall entity named FTLD [1,5]. All these syndromes were recognised to overlap throughout disease progression, and it was recently demonstrated that there is a convergence of the syndromes in the course of the disease [9].

Brain imaging techniques have been successfully used to characterise the different syndromes, allowing us to explore the involved neural systems, and helping us in comprehend their pathogenetic mechanisms [6,10,11]. The question that remains opens, however, is whether a single neural network underscoring FTLD or different entities might be discriminated by structural or functional neuroimaging. The further problem in evaluating FTLD patients is that, in most cases, more than one behavioural or cognitive symptom occurs in the same patient [12]. In addition, the use pre-defined clinical criteria aimed to verify such imaging data may imply a risk of circularity.

At present, clinical clusters might provide the opportunity to identify specific cognitive and behavioural patterns beyond current clinical criteria.

With this aim in mind, we applied Latent Profile Analysis (LPA), a latent variable model that is used to detect subgroups or syndromes based on the observed relationship between chosen continuous indicators or symptoms [13]. LPA is here employed to rationalise the large clinical and neuropsychological data set with an open approach, allowing us to reveal discrete clinical presentation.

Once the data-driven classification has been formed, its coherence was verified by functional neuroimaging information.

## Methods

### Subjects

This study is part of an ongoing research program aimed at evaluating the core feature of FTLD. Patients who met the Neary [14] and McKhann [15] criteria for FTLD were consecutively recruited from March 2003 through July 2005 from the "Center for Aging Brain and Neurodegenerative Diseases", University of Brescia, Italy.

All subjects underwent a somatic and neurological evaluation, and routine laboratory examination, a brain structural Magnetic Resonance Imaging (MRI) study, and a brain functional Single Photon Emission Tomography (SPECT) study.

The diagnostic assessment involved a review of full medical history, a semi-structured neurological examination, and a complete mental status evaluation by two independent and experienced reviewers (B.B., A.P.). Only patients with full consensus agreement by the reviewers were enrolled.

Motor impairment was evaluated using the motor subscale of Unified Parkinson Disease Rating Scale (UPDRS) III [16]. Global cognitive function assessment was made according to a standardized battery, including the Mini-Mental State Examination (MMSE) [17]. The neuropsychological assessment was made with the following tests: Raven Colored Progressive Matrices [18], Controlled Oral Word Association Test and Category Fluency [19], Clock Drawing Test [20], Rey Complex Figure Copy and Recall [21], Story Recall Test [22], Digit Span [23], Trail Making Test A and B [24], Token Test [25], and De Renzi Imitation Test [26]. Instrumental Activities of Daily Living (IADL) [27], and Basic Activities of Daily Living (BADL) [28] were assessed as well. Behavioural and psychiatric disturbances were evaluated by Neuropsychiatry Inventory (NPI) [29], and Frontal Behavioual Inventory (FBI) [30].

Patients considered to have a positive family history were those who had a first-degree relative with dementia, Parkinsonism, or motor neuron disease. No patients belonging to the same family were included.

All participants were made fully aware of the research goals, and the signature of an informed consent was required from all subjects. The work was conducted in accordance with local clinical research regulations and in conformity with the Helsinki Declaration.

### Inclusion criteria

The Neary [14] and McKhann criteria [15] for FTLD were fulfilled by all subjects. In particular, bvFTD was defined by character change and disorder of social conduct. SD was defined by a prominent comprehension disorder (impaired understanding of word meaning and/or object identity), difficulty in naming, and asking for the name of nouns and objects. PNFA was diagnosed when the first symptom was an isolated disorder of expressive language when other aspects of cognition and daily living functions were relatively well preserved. CBD exhibited unilateral rigidity and apraxia, some of them having 'alien hand'. In these patients the extrapyramidal syndrome developed first and was followed by cognitive changes. PSP showed vertical gaze palsy, repetitive falls, axial rigidity and pseudobulbar palsy; a poor response to L-dopa treatment was counted as an additional inclusion criterion.

In FTLD patients, an adjunctive inclusion criterion was an initial diagnosis confirmation after the patient had been followed periodically for a 1.5 year period.

### Exclusion criteria

Stringent exclusion criteria were applied as follows: a) cerebrovascular disorders, previous stroke, hydrocephalus, and intra-cranial mass documented by MRI; b) a history of traumatic brain injury or another neurological disease (e.g. seizures, choreoathetosis, cerebellar ataxia); c) another extrapyramidal syndrome (e.g. Parkinson disease, Lewy Body Disease, Vascular Parkinsonism, Multiple Systemic Atrophy) according to current clinical criteria; d) significant medical problems (e.g. poorly controlled diabetes or hypertension; cancer within the past 5 years; clinically significant hepatic, renal, cardiac, or pulmonary disorders); e) major depressive disorder, bipolar disorder, schizophrenia, substance abuse disorder, or mental retardation according to criteria of the DSM-IV.

### ^99m^Tc-ECD SPECT acquisition

For brain functional data comparisons, a group of healthy subjects (n = 15, mean age ± SD = 56.3 ± 15.4) were recruited among patients' spouses or relatives and were included as normal controls. They were interviewed, assessed for neurological or cognitive dysfunction, evaluated for diseases that were exclusion criteria for the patients group, and underwent a brain SPECT study.

Both patients and controls were administered an intravenous injection of 1110 MBq ^99m^Tc-ECD (ethylcysteinate dimer, Neurolite, Bristol-Myers Squibb Pharma) in a rest condition, lying supine in a quiet, dimlylit room.

All subjects were imaged using a dual-head rotating gamma camera (VG MILLENIUM GE) fitted with a low energy, high-resolution collimator, 30 minutes after intravenous injection of ^99m^Tc-ECD.

A 128 × 128 pixel matrix was used for image acquisition with 120 views over a 360° orbit (in 3° step) with a pixel size and slice thickness of 1 mm, in 27 min or more if total counts were lower than 5 × 10^6^. Image reconstruction was performed by a ramp filtered-back projection and three-dimensionally smoothed with a Metz filter (order 3, enhancement 1.24, FWHM 6.7 mm, cut-off 0.61 cycles cm^-1^). The reconstructed images were corrected for gamma ray attenuation using the Chang method (attenuation coefficient: 0.11 cm^-1^).

### Image pre-processing and analysis

Statistical Parametric Mapping (SPM2, Welcome Department of Cognitive Neurology, University College, London), and Matlab 6.1 (Mathworks Inc., Sherborn, MA) were used for image pre-processing. Images were spatially normalized to a reference stereotactic template (Montreal Neurological Institute, MNI), and smoothed by a Gaussian kernel of 8 × 8 × 8 mm FWHM SPECT data analysis was performed in blind to clinical and genetic diagnosis.

Global differences in the distribution, and the effect of age on the tracer's uptake were covariated out for all voxels. Comparisons across the different groups were made using *t*-statistics with appropriate linear contrasts [31]. We considered any cluster above a statistical threshold set at *P *< 0.001.

We performed the following group comparisons: (1) each FTLD group, i.e. bvFTD, SD, PNFA, PSP and CBD *vs*. controls, in order to explore the hypoperfusion patterns according to clinical diagnosis, and (2) FTLD patients belonging to each Latent Class group *vs*. controls, to explore the hypoperfusion patterns according to latent classes.

### Statistical analysis

Latent Profile Analysis (LPA) was used to determine the number and composition of groups in which participants aggregated on the basis of their indicator values.

LPA, as Latent Class Analysis (LCA), is a latent variable model that serves to cluster subjects. LCA explains the clustering of cases, based on the relationship of a set of observed (manifest) **categorical **indicators by assuming that the patterns of values are determined by a latent (unobserved) categorical variable. Inversely, LPA allows **continuous **indicators to be present. For both, the number of categories of the latent variables (i.e. number of latent classes) represents the number of different clusters in subjects. In the classic form of the LPA used here, observed continuous indicators within each latent class were assumed to be uncorrelated. In other words, the model supposes that the correlation matrix between continuous outcomes is due to unobserved heterogeneity, i.e. the pairwise relationships between the continuous outcomes are due to mixing an unknown number of different classes of individuals, each having unrelated outcomes. This is also referred as **conditional independence **assumption, with the idea that if a sufficient number of classes is introduced, the conditional independence is more likely to hold. Thus, the latent categorical variable captures population heterogeneity, and the parameters of the model are: latent class prevalences, indicator means within latent classes, and indicator variances common to all latent classes.

We performed a sequence of five LPA models, from one to five classes, with varying across-class indicator means, and across-class common indicator variances. Neuropsychological performances (MMSE, Raven Colored Progressive Matrices, Controlled Oral Word Association Test, Category Fluency, Clock Drawing Test, Rey Complex Figure Copy and Recall, Story Recall Test, Digit Span, Trail Making Test A and B, Token Test, and De Renzi Imitation Test), functional impairment (IADL, BADL), and behavioural disturbance (NPI, FBI) and motor impairment (UPDRS-III) were introduced in the LPA as observed continuous indicators. UPDRS-III and De Renzi Imitation Test were excluded because they resulted uncorrelated with the previous variables in a preliminary descriptive data analysis.

Model parameters were estimated using Maximum Likelihood Estimator (MLE) based on the Expectation Maximization (EM) algorithm. The Akaike's Information Criterion (AIC = -2 × model log-likelihood + 2 × number of model parameters) and Bayesian Information Criterion (BIC = -2 × model log-likelihood + log(*n*) × number of model parameters) were computed to compare such competing models, and the selected model was the one minimizing either AIC or BIC [32].

Using the entropy index, the quality of the resulting classification was also evaluated in terms of the separation of the latent classes. Entropy denotes how possible it is to predict class membership given the observed indicators. Values range from 0 to 1, and high values (>0.90) indicate that the latent classes are highly discriminative.

The statistical significance of the parameter estimates were evaluated by t-tests (= parameter/standard error) considering robust standard errors. Multiple comparisons procedures based on the Fisher's Least Significant Difference (LSD) testing of across-class mean estimates were used to select discriminative indicators. The significance level was established at P < 0.05, two-sided.

All models were fit using Mplus software (version 3.13 for Windows), and full details of the computing approach can be found in Muthèn & Muthèn user's guide [33].

## Results

### Subjects

Ninety-seven patients entered the study. Demographic and clinical characteristics according to clinical diagnosis are reported in Table 1. Overall, FTLD patients were mild for global cognitive decline. BvFTD diagnosis was the most prevalent (42.2%), SD and PNFA diagnoses were less prevalent (5–6%). BvFTD showed higher behavioural disturbances (NPI and FBI-AB) compared to SD and PNFA patients, who had a different pattern of behavioural disturbances (as illustrated by high loadings in FBI-A but low scores in FBI-B); whilst CBD and PSP, as expected, showed the worst motor impairment.

Neuropsychological profile in bvFTD, SD, PNFA, CBD, and PSP patients, and the number of patients with pathological scores in each group, is reported in Table 2. Neuropsychological tests which resulted pathological in more than 50% of cases, according to Italian normative data, are highlighted: bvFTD showed greater behavioural disturbances, and SD were more impaired in verbal and non-verbal memory (Short Story and recall of Rey Complex Figure), category fluency, and executive functions (Trail Making B); PNFA patients, as expected, had language deficits (verbal and category fluency) and were more impaired in visuo-spatial skills (Trail Making A); while CBD and PSP showed deficits in visuo-spatial skills (copy of Rey Complex Figure and Trail Making A). Pathological scores of the De Renzi Imitation test were found in 43.8% of CBD patients because of the mild stage of the disease.

### Functional correlates of clinical diagnosis

**Figure 1:**
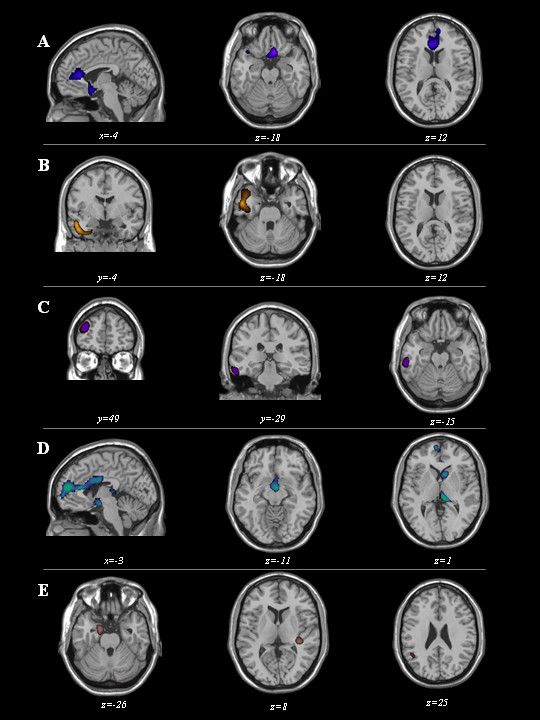
**Maps of significant voxels representing regions of hypoperfusion in FTLD patients according to clinical diagnosis, superimposed to reference T1-weighted MRI image**. bvFTD: behavioural variant of Frontotemporal Dementia (first row), SD: Semantic Dementia (second row), PNFA: Progressive Non-Fluent Aphasia (third row), PSP: Progressive Supranuclear Palsy (fourth row), and CBD: Corticobasal Degeneration (fifth row) patient subgroups are reported. Statistical threshold, P < 0.001, T ≥ 3.99, minimum cluster size = 50 voxels. Neurological convention: left is on the left side and *vice versa*.

SPM2 was applied to SPECT scans, which were subgrouped according to clinical diagnosis (total number = 50; bvFTD = 20, SD = 3, PNFA = 2, CBD = 11, and PSP = 14). As reported in Figure 1, bvFTD showed hypoperfusion in medial frontal cortex (x, y, z = 6, 16, -12; T = 4.35, cluster size = 845; and -4,32,12; T = 3.97, cluster size = 834) and left temporal cortex (-16,12,-36; T = 3.92, cluster size = 347); SD patients had significant hypoperfusion in left temporal pole (-44,-4,-32; T = 4.87, cluster size = 1162); PNFA showed an involvement of both left frontal cortex (-32,54,20; T = 4.42, cluster size = 230) and left inferior temporal gyrus (-58,-32,-20; T = 4.09, cluster size = 167); PSP patients had significant involvement in medial frontal cortex (-2,30,16; T = 4.23, cluster size = 3846), dorsolateral frontal cortex (-48,18,24; T = 4.41, cluster size = 226) and basal ganglia and mesencephalon; CBD patients showed hypoperfusion in left inferior parietal lobe (-46,-52,28; T = 3.49, cluster size = 160), right superior temporal gyrus (36,-30,8; T = 3.77, cluster size = 141), and left uncus and parahippocampal region (-18,2,-32, T = 4.02; cluster size = 312).

### Latent Profile Analysis

LPA models were fitted with 16 variables, that had from 1 to 5 LCs. Among 97 FTLD patients, 5 were excluded because of missing values. The fit statistics suggested that the model with 4 LCs had the best fit, producing minimum AIC and BIC values, and having the highest separation of the LCs evaluated by entropy measure (see Table 3). The second best model was the LPA model with 3 LCs. This model was preferred because the estimated mean profiles were more interpretable than those with 4 LCs. Additionally, the reduction in BIC statistic from 3 to 4 classes was close to zero [BIC(4) = 5401 vs. BIC(3) = 5407], and the entropy measures were close to 1 and comparable [entropy (4) = 0.963 vs. entropy (3) = 0.951].

Table 4 shows the parameter estimates for the 3 selected LCs. These parameters represent the latent classes' prevalences, the latent class specific mean profiles and the common variances of 16 variables considered in the LPA model.

Of the total sample, 18 (18.5%) patients belonged to Latent Class 1 (LC1), 41 (42.3%) to Latent Class 2 (LC2), and 38 (39.2%) to Latent Class 3 (LC3).

**Figure 2:**
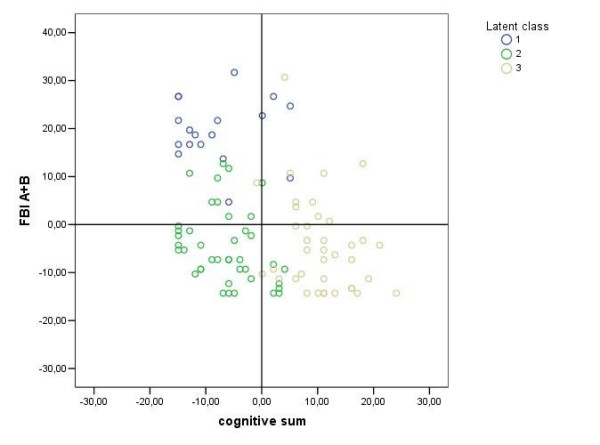
**Scatter-plot of FTLD patients labelled according to generated three Latent Classes**. y axis: values of behavioural disturbances (measured by Frontal Behavioural Inventory Scale, FBI-A plus FBI-B,); x axis: values of cognitive deficits (cognitive sum, i.e. the sum of Equivalent Scores of all the neuropsychological tests included in the assessment, the higher the value the better the performance; see Table 4). The centroid (0;0) represents the overall mean profile; compared to the average value of the whole sample, LC1 patients have higher cognitive sum values (i.e. less severe cognitive impairment); while LC2 and LC3 patients have lower cognitive sum values (i.e. more severe cognitive impairment); *vice versa *the behavioural deficit values are higher in the LC1 than in the LC2 and LC3 patients.

As shown in Figure 2, these three LCs were significantly separated from each other.

Using statistically significant pairwise mean differences provided by multiple comparisons procedure which was based on the Fisher's Least Significant Difference (LSD) testing, the latent classes are described as follows: LC1 denoted subjects with high means on behavioural disturbances (FBI-A, FBI-B, and NPI) and daily living functional impairment (BADL, IADL) compared to the other two classes. LC3 was defined by subjects with fewer cognitive disturbances measured by this neuropsychological assessment compared to those in LC1 and LC2. Finally, LC2 represented the patients with fewer behavioural disturbances but an equal cognitive profile compared to LC1 patients. In addition, LC2 shared identical behavioural deficits, but have more pronounced cognitive impairment than LC3 patients.

In clinical terms, behavioural disturbances, such as disinhibition and abnormal social conduct (as demonstrated by high FBI-B scores), characterized LC1 patients. LC2 patients showed a profile of substantial cognitive impairment with in particular a poor performance in the Trail Making B test. LC3 patients showed less cognitive impairment than the other two classes, and behavioural disturbances similar to LC2 patients. In order to better define the behavioural aspect of LC3 patients, we therefore considered the subscores of the behavioural profile. We found that LC3 and LC2 mainly differed for a greater prevalence of depressive symptoms in the former (data not shown).

Thus, we adopted the following clinical labels for the 3 LCs: "pseudomanic behaviour" endophenotype for LC1 and "pseudodepressed behaviour" endophenotype for LC3 (see reference 34); LC2 was named as "cognitive" endophenotype.

### Functional correlates of Latent Classes

Functional SPECT data were then analysed according to the LCs. Fifty patients out of 97 underwent a SPECT scan. Nine patients belonging to LC1, 17 belonging to LC2, and 24 belonging to LC3 were compared to control subjects, respectively.

**Figure 3:**
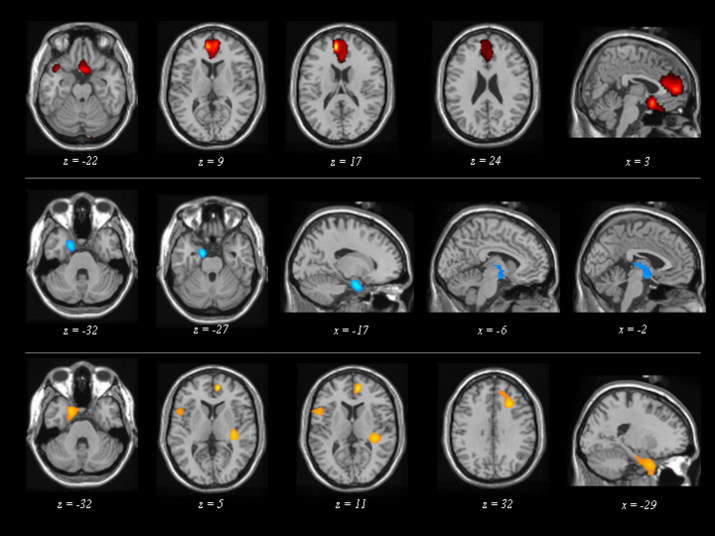
**Maps of significant voxels representing regions of hypoperfusion in FTLD patients according to the three Latent Classes, superimposed to reference T1-weighted MRI image**. LC: Latent Class. LC1 (first row), LC2 (second row), and LC3 (third row) patient subgroups are reported. Statistical threshold, P < 0.001, T ≥ 3.40, minimum cluster size = 50 voxels. Neurological convention: left is on the left side and *vice versa*.

As shown in Figure 3, distinct functional patterns were found in LC1, LC2, and LC3 subgroups.

Comparisons of LC1 ("pseudomanic behaviour" endophenotype) *vs*. controls showed significant hypoperfusion in medial frontal cortex (x, y, z = -8, 50, 16; T = 5.19, cluster size = 1167), in the orbitobasal frontal cortex (0,12 -16; T = 4.52, cluster size = 871), and in left temporal pole (-38,14,-24; T = 3.82, cluster size = 171).

LC2 ("cognitive" endophenotype) *vs*. controls showed comparable hypoperfusion in the left temporal pole (-14,-4,-24; T = 4.76, cluster size = 457) and in subcortical brain region (-2,-6,-12; T = 4.76, cluster size = 457).

LC3 ("pseudodepressed behaviour" endophenotype) showed significant hypoperfusion in left temporal pole (-18,8,-36; T = 4.31, cluster size = 807), right dorsolateral frontal cortex (10,52,8; T = 4.86, cluster size = 506), and insula (-54,10,16; T = 4.18, cluster size = 256; 36,-30,8; T = 4.45, cluster size = 275; 42,4,0; T = 3.56, cluster size = 51).

### Clinical diagnosis and Latent Class comparisons

Finally, LCs were compared to the clinical diagnosis.

**Figure 4:**
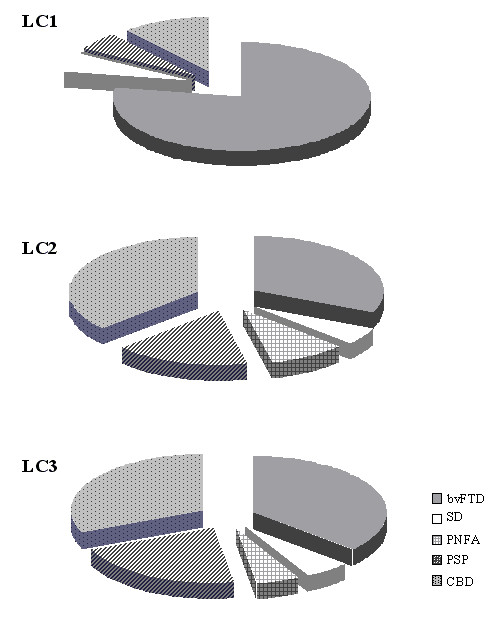
**Proportion of each clinical diagnosis across the three Latent Classes**. LC: Latent Class; bvFTD: behavioural variant of Frontotemporal Dementia, SD: Semantic Dementia, PNFA: Progressive Non-Fluent Aphasia, PSP: Progressive Supranuclear Palsy, and CBD: Corticobasal Degeneration.

In Table 5 and Figure 4, the analysis of proportion of clinical diagnoses within LCs was reported. bvFTD patients mainly belong to LC1 (the pseudomanic behaviour endophenotype), but there was a high overlap of different clinical diagnoses in LC2 and LC3.

## Discussion

The aim of this study was to establish a data-driven nosology and to provide the neuroimaging correlates in FTLD. This analysis suggested a division into three groups, clearly distinguished by specific functional hypoperfusion patterns.

The clinical, pathological and genetic heterogeneity of FTLD accounts for its present nosological controversy, and opens the question of whether this generic label represents a unique syndrome best viewed as complex, or if it includes different and distinct entities.

At present, FTLD classification relies upon clinical symptoms at disease presentation, but the convergence of the different clinical pictures in overlapping syndromes and their poor concordance with pathology, made of the FTLD clinical diagnosis a challenging problem [9]. We currently differentiate FTLD in bvFTD, SD, PNFA, PSP, CBD, and these clinical distinctions may also have functional correspondences (see Figure 1). The mismatch, however, between clinical pictures and pathology makes the present classification largely unsatisfactory. This suggests the need of an alternative explanation to describe FTDL discrete clinical presentations.

In this study, we tried to disentangle these issues, employing the open statistical approach provided by Latent Profile Analysis and applying it to a large sample of FTLD patients. Our aim was to single out which, and how many clinical presentations could be generated by a statistical analysis blinded to an extended cognitive/behavioural data set in FTLD patients.

Latent Profile Analysis posits that a heterogeneous group can be reduced to several homogeneous subgroups through evaluating and then minimizing the pairwise correlations among responses across multiple continuous variables. Thus, Latent Profile Analysis is capable of determining the number and composition of unobserved latent classes that produce observed data. This approach is particularly useful when there is evidence that certain symptoms co-aggregate at above normal levels (that is, symptoms that are beyond what is usually seen in patients who present certain syndrome patterns) to form distinct clusters.

In the present study, cognitive performance, the severity of behavioural symptoms, and functional impairment were examined in a large sample of FTLD patients, and three distinct clusters were detected, named "pseudomanic behaviour", "cognitive" and "pseudodepressed behaviour".

The first, i.e. LC1, was represented by greater behavioural disturbances, such as dishinibition and abnormal social conduct, as evidenced in FBI and NPI scores. The concomitant impairment in BADL and IADL in these patients is therefore the result of inadequacy or disinhibition rather than the consequence of the cognitive decline. The second LC2 profile was underscored by a "cognitive" endophenotype, mainly characterized by executive dysfunction. The third LC3 showed better performances in neuropsychological test scores compared to the other two LCs, and subtle behavioural abnormalities, mainly represented by depressive symptoms.

It is noteworthy to mention that motor impairment, measured by UPDRS-III scale, was not used to define the latent classes, and was uncorrelated with either cognitive or behavioural profiles, suggesting an independence between motor aspects and cognitive/behavioural performances.

Thus, referring to the nosology of FTLD, our data-driven approach identified three clinical syndromes. In the same vein, a recent experimental work applied a similar approach, i.e. Factor Analysis, to clarify the classification issue in Progressive Aphasia [2].

As a second goal of the present study, the SPECT imaging modality and Statistical Parametric Mapping (SPM2) were used to identify functional correspondence of the FTLD as generated by Latent Profile Analysis. The SPM2 method was applied to verify if the generated LCs depended on different stage of the disease, or if they were the expression of three different endophenotypes.

The three clusters obtained by Latent Profile Analysis were underscored by specific functional correlates. Hypoperfusion in frontal medial and orbitobasal cortex was found in the "pseudomanic behaviour" endophenotype, subcortical brain region hypoperfusion was detected in the "cognitive" endophenotype, hypoperfusion in the dorsolateral frontal cortex and insula characterised the "pseudodepressed behaviour" endophenotype (Figure 3). These data provide evidence that specific neurofunctional-symptom cluster relationship can be delineated in patients with FTLD, thus excluding that these latent classes represent different disease stages.

Interestingly, the pseudomanic and pseudodepressed endophenotypes paralleled previous behavioural and metabolic findings reported in a group of FTLD patients [34]. The main subset of symptoms in "cognitive" endophenotye, such as executive dysfunctions, can well be attributed to impairment of the dorsolateral-frontal circuit at the basal ganglia level; in fact, when data were explored at a lower threshold (P < 0.005), not only subcortical brain regions, but also dorsolateral frontal cortex was involved (-32,58,16; T = 2.87, cluster size = 101).

An increasing number of studies evaluating brain tissue volume and metabolic function in dementia demonstrate that regional tissue loss, or hypometabolism, correlates with specific cognitive or behavioural impairment in FTLD [35,4]. FTLD patients, however, had profuse cognitive and behavioural abnormalities, thus suggesting that all these symptoms could have had a common neurofunctional basis [36]. To date, few studies in either dementia or focal lesions has systematically examined the functional correlates of clustered behavioural and neuropsychological symptoms, such as here conducted by Latent Profile Analysis, instead of individual symptoms. We also performed the SPM2 analysis on the basis of pre-defined criteria (see Figure 1), confirming previous neuroimaging findings [7,10,11].

The present results suggest that FTLD syndromes lie on a continuum rather then existing as independence entities. Latent Profile Analysis approach demonstrated that FTLD can be summarised into three different clinical categories with a poor concordance with the usual clinical classification. Indeed, despite of a higher level of concordance in diagnosis distribution between LC1 and bvFTD, the other two LCs, i.e. LC2 and LC3, are otherwise variably represented (see Figure 4).

It has been recently reported that there is a convergence of the syndromes in the course of the disease [9]. In the future, it would be interesting to evaluate the clinical and overlapping aspects of these clusters over time.

Notwithstanding, approaches based on standardised assessment, similar or different to those presented in this study, are crucial for a clear-cut description of the wide range of features, both behavioural and cognitive, that may characterise FTLD and related disorders.

The results of this study should be replicated in other sample size cross-sectional studies, and these should be performed to determine if the same endophenotypes remain stable across studies. We indeed acknowledge the need of prognostic outcomes to prove the usefulness of the present scheme. Moreover, neuropathological confirmation would be necessary to further understand the relationship between clinical and neuropathological endophenotypes. In an era of treatment that targets disease-mechanism, it would be desirable that Latent Profile Analysis will be used to further discriminate molecular/genetic determinants of FTLD pathology.

## Conclusion

In conclusions, among the mild FTLD variants, characterised by a wide clinical overlap, specific clinical and functional neural networks may be identified at the beginning of the disease. Further longitudinal studies will be devoted to analysing the evolution of such clusters in order to delineate the pattern of disease progression.

## Abbreviations

FTLD: Frontotemporal Lobar Degeneration; SPECT: Single Photon Emission Tomography; bvFTD: behavioural variant of frontotemporal dementia; SD: Semantic Dementia; PNFA: Progressive Non-fluent Aphasia; PSP: Progressive Supranuclear Palsy; CBD: Corticobasal Degeneration; LPA: Latent Profile Analysis.

## Competing interests

The author(s) declare that they have no competing interests.

## Authors' contributions

BB conceived of the study, and participated in its design and coordination and in the draft of the manuscript; MG participated in the design of the study, performed statistical analysis and revised the manuscript critically for intellectual content; DP participated in the design of the study, in neuroimaging analyses and revised the manuscript critically for intellectual content; CA and AA performed the clinical evaluation of the patients and have made substantial contribution to interpretation of data; BP has made substantial contributions in the acquisition of the data; MDL has been involved in the study design and in revising it critically for important intellectual content; AP conceived of the study, and participated in its design and in coordination and drafted the manuscript. All authors read and approved the final manuscript.

## Pre-publication history

The pre-publication history for this paper can be accessed here:



## Figures and Tables

**Table 1:** Demographic and clinical characteristics of included FTLD patients.

***Diagnosis***	**bvFTD**	**SD**	**PNFA**	**CBD**	**PSP**
N	41	5	6	16	29
Age, years	66.8 ± 7.2	69.0 ± 9.0	61.3 ± 7.3	61.6 ± 7.7	72.7 ± 5.2
Gender, F%	48.8 (20)	80 (4)	83.3 (5)	50 (8)	41.4 (12)
FH*	36.4 (12)	0 (0)	40 (2)	18.3 (3)	23.1 (6)
Education, years	7.1 ± 3.0	10.0 ± 3.7	6.8 ± 3.2	8.5 ± 4.8	7.2 ± 4.5
Age at onset, years	63.7 ± 7.8	67.2 ± 9.1	56.6 ± 3.2	61.1 ± 5.4	70.5 ± 4.9
UPDRS-III	9.2 ± 8.7	3.2 ± 3.1	6.1 ± 6.6	18.1 ± 10.9	22.7 ± 11.8
MMSE	21.6 ± 5.8	21.0 ± 6.4	18.6 ± 6.8	22.6 ± 4.5	24.7 ± 4.0
NPI	19.9 ± 14.1	12.0 ± 9.7	15.1 ± 10.5	8.0 ± 9.2	9.8 ± 9.8
FBI-A	12.0 ± 7.7	11.8 ± 8.1	13.6 ± 8.9	8.0 ± 8.4	4.8 ± 6.5
FBI-B	8.1 ± 6.7	4.8 ± 4.4	4.5 ± 5.3	1.6 ± 2.1	2.4 ± 3.9
FBI-AB	21.8 ± 15.5	16.6 ± 10.7	19.0 ± 15.0	13.6 ± 8.6	7.3 ± 8.8
BADL	0.7 ± 1.5	0.4 ± 0.8	0.0 ± 0.0	0.5 ± 0.9	0.8 ± 1.4
IADL	2.3 ± 2.7	2.2 ± 2.6	1.6 ± 2.6	0.7 ± 1.5	1.3 ± 2.0

bvFTD: behavioural variant of Frontotemporal Dementia; SD: Semantic Dementia; PNFA: Slowly Progressive Aphasia; CBD: Corticobasal Degeneration; PSP: Progressive Supranuclear Palsy; N: number; F: female; FH: Family History; UPDRS: Unified Parkinson Disease Rating Scale; MMSE: Mini-Mental State Examination; NPI: Neuropsychiatry Inventory; FBI: Frontal Behavioural Inventory; BADL: Basic Activities of Daily Living; IADL: Instrumental Activities of Daily Living.

Number of subjects between brackets. *missed values are due to unknown FH.

**Table 2:** Neuropsychological assessment in FTLD patients.

***Diagnosis***	**BvFTD**	**SD**	**PNFA**	**CBD**	**PSP**
Short story	8.3 ± 4.7 (41.4)	5.5 ± 1.3 (80.0)	5.1 ± 3.4 (83.3)	8.5 ± 4.9 (31.5)	9.7 ± 2.9 (47.3)
Raven Colored Progressive Matrices	21.7 ± 5.7 (26.8)	29.2 ± 5.2 (0.0)	26.1 ± 3.8 (0.0)	23.0 ± 6.0 (25.0)	24.3 ± 6.4 (31.5)
Rey Complex Figure, Copy	23.5 ± 10.5 (48.7)	26.5 ± 12.9 (40.0)	34.3 ± 1.1 (0.0)	22.1 ± 10.5 (68.7)	24.5 ± 9.2 (64.7)
Rey Complex Figure, Recall	11.6 ± 7.2 (43.9)	11.4 ± 3.0 (60.0)	16.0 ± 1.3 (0.0)	12.0 ± 5.6 (56.2)	14.9 ± 6.8 (63.1)
Verbal fluency	22.1 ± 11.1 (34.1)	19.6 ± 10.0 (40.0)	10.5 ± 1.7 (66.6)	23.4 ± 11.5 (31.2)	23.8 ± 9.3 (26.3)
Category Fluency	25.5 ± 11.2 (48.7)	18.0 ± 10.2 (60.0)	23.7 ± 13.7 (50.0)	32.1 ± 10.8 (18.7)	30.5 ± 6.7 (26.3)
Digit Span	5.5 ± 1.5 (4.8)	5.7 ± 0.7 (0.0)	4.0 ± 2.3 (16.7)	5.2 ± 1.1 (6.2)	5.7 ± 0.9 (0.0)
Token Test	28.4 ± 5.6 (2.1)	25.3 ± 3.1 (40.0)	26.3 ± 2.2 (16.0)	28.9 ± 5.5 (25.0)	32.2 ± 3.0 (10.5)
Trail Making A	158.8 ± 171.2 (39.0)	224.4 ± 235.6 (40.0)	309.1 ± 220.9 (66.6)	130.9 ± 154.6 (31.2)	123.6 ± 114.4 (63.1)
Trail Making B	275.0 ± 151.9 (39.0)	285.8 ± 165.6 (60.0)	281.3 ± 149.8 (50.0)	292.5 ± 175.7 (43.7)	251.3 ± 145.9 (47.3)
De Renzi Imitation Test	66.2 ± 8.2 (9.7)	71.3 ± 1.1 (0.0)	62.5 ± 10.6 (33.3)	53.7 ± 16.1 (43.8)	66.6 ± 5.2 (0.0)

bvFTD: behavioural variant of Frontotemporal Dementia; SD: Semantic Dementia; PNFA: Progressive Non-Fluent Aphasia; CBD: Corticobasal Degeneration; PSP: Progressive Supranuclear Palsy. Percentage of subjects with pathological scoring according to Italian normative data between brackets (percentage > 50% are underlined)

**Table 3:** Goodness of fit statistics, and class frequencies for LPA models from 1 to 5 latent classes.

	**LC (1)**	**LC (2)**	**LC (3)**	**LC (4)**	**LC (5)**
**Log-L (H0)**	-29663.70	-2636.05	-2554.08	-2512.92	-2507.12
**n parameters**	32	49	66	83	100
**AIC**	5991.4	5370.1	5240.2	5191.8	5214.2
**BIC**	6073.8	5493.7	5406.6	5401.1	5466.4
**Entropy**		0.92	0.951	0.963	0.945

**Class frequencies (%)**					

**n1**	92 (100)	44 (47.8)	17 (18.5)	8 (8.7)	28 (30.4)
**n2**		48 (52.2)	38 (41.3)	12 (13.0)	3 (3.3)
**n3**			37 (40.2)	33 (35.9)	17 (18.5)
**n4**				39 (42.4)	29 (31.5)
**n5**					15 (16.3)

LPA: Latent Profile Analysis; LC: Latent Class; Log-L (H0): Log-likelihood of hypothesized model (H0)

AIC: Akaike's Information Criterion (= -2 × model log-likelihood + 2 × number of model parameters); BIC: Bayesian Information Criterion (= -2 × model log-likelihood + log(n) × number of model parameters)

**Table 4:** Maximum Likelihood estimates (MLE) of the mean profiles, and common of the clinical and neuropsychological assessment variables across the selected three LCs (*)

	***Latent class means (#)***			
		
***Variable***	***LC1 (n = 18)***	***LC2 (n = 41)***	***LC3 (n = 38)***	***Common variances***
BADL (lost functions)	**2.444**	0.366	0.184	1.049
IADL (lost functions)	**5.111**	1.244	0.632	2.858
NPI	**31.556**	8.707	11.211	0.847
FBI, A	**19.833**	7.610	6.289	4.025
FBI, B	**14.056**	2.122	3.711	1.524
*Neuropsychological Assessment*				
MMSE	19.283	20.917	25.790	2.198
Short Story (ES)	0.889	0.537	1.632	1.551
Colored Progressive Matrices (ES)	0.722	0.683	2.632	0.807
Rey Figure, Copy (ES)	0.278	0.585	2.211	2.089
Rey Figure, Recall (ES)	0.333	1.000	2.895	2.116
Fluency, Letter (ES)	1.000	1.000	2.184	2.122
Fluency, Category (ES)	0.222	0.756	2.632	1.279
Digit PNFAn (ES)	2.111	2.220	3.211	2.466
Token Test (ES)	0.722	0.927	2.895	1.682
Trial-Making, Test A (ES)	0.278	0.463	3.158	1.165
Trial-Making, Test B (ES)	0.278	0.366	2.105	1.226

BADL: Basic Activities of Daily Living; IADL: Instrumental Activities of Daily Living; NPI: Neuropsychiatry Inventory; FBI: Frontal Behavioural Inventory. ES: Equivalent Scores (according to Italian normative data, range 0–4; 0 = poor performance to 4 better performance). (*) All MLEs were statistically significant (i.e different to zero using t-test: P < 0.05), and expressed mean values.

(#) Statistically significant (P < 0.05) means differences between LC1 vs. (LC2, LC3) are bold-underline, while between (LC1, LC2) vs. LC3 are underline.

**Table 5:** FTLD diagnosis distribution according to Latent Classes.

**LC/Diagnosis**	***BvFTD ***(*n *= 41)	***SD ***(*n *= 5)	***PNFA ***(*n *= 6)	***CBD ***(*n *= 16)	***PSP ***(*n *= 29)
**LC1 **(n = 18)	77.8 (14)	5.6 (1)	0.0 (0)	5.6 (1)	11.2 (2)
**LC2 **(n = 41)	31.7 (13)	4.9 (2)	9.8 (4)	17.1 (7)	36.6 (15)
**LC3 **(n = 38)	36.8 (14)	5.3 (2)	5.3 (2)	21.1 (8)	31.5 (12)

TOTAL	42.3 (41)	5.2 (5)	6.2 (6)	16.5 (16)	29.9 (29)

LC: Latent Class; bvFTD: behavioural variant of Frontotemporal Dementia; SD: Semantic Dementia; PNFA: Slowly Progressive Aphasia; CBD: Corticobasal Degeneration; PSP: Progressive Supranuclear Palsy.

Results are expressed as conditional Latent Classes percentage and number of patients between brackets.
